# Advances in molecular targeted therapies to increase efficacy of (chemo)radiation therapy

**DOI:** 10.1007/s00066-023-02064-y

**Published:** 2023-04-11

**Authors:** Kristina Viktorsson, Thorsten Rieckmann, Maximilian Fleischmann, Markus Diefenhardt, Stephanie Hehlgans, Franz Rödel

**Affiliations:** 1 Department of Oncology/Pathology, Karolinska Institutet, Visionsgatan 4, 17164 Solna, Sweden; 2 Department of Radiation Oncology, University Medical Center Hamburg Eppendorf, Martinistraße 52, 20246 Hamburg, Germany; 3 Department of Otolaryngology, University Medical Center Hamburg Eppendorf, Martinistraße 52, 20246 Hamburg, Germany; 4 Department of Radiotherapy and Oncology, Goethe University, Theodor-Stern-Kai 7, 60590 Frankfurt am Main, Germany; 5 Frankfurt Cancer Institute (FCI), University of Frankfurt, Theodor-Stern-Kai 7, 60590 Frankfurt, Germany; 6 German Cancer Consortium (DKTK) partner site: Frankfurt, Im Neuenheimer Feld 280, 69120 Heidelberg, Germany

**Keywords:** Radiation sensitization, Translational research, DNA damage response, Noncoding RNAs, Nanoparticles

## Abstract

Recent advances in understanding the tumor’s biology in line with a constantly growing number of innovative technologies have prompted characterization of patients’ individual malignancies and may display a prerequisite to treat cancer at its patient individual tumor vulnerability. In recent decades, radiation- induced signaling and tumor promoting local events for radiation sensitization were explored in detail, resulting the development of novel molecular targets. A multitude of pharmacological, genetic, and immunological principles, including small molecule- and antibody-based targeted strategies, have been developed that are suitable for combined concepts with radiation (RT) or chemoradiation therapy (CRT). Despite a plethora of promising experimental and preclinical findings, however, so far, only a very limited number of clinical trials have demonstrated a better outcome and/or patient benefit when RT or CRT are combined with targeted agents. The current review aims to summarize recent progress in molecular therapies targeting oncogenic drivers, DNA damage and cell cycle response, apoptosis signaling pathways, cell adhesion molecules, hypoxia, and the tumor microenvironment to impact therapy refractoriness and to boost radiation response. In addition, we will discuss recent advances in nanotechnology, e.g., RNA technologies and protein-degrading proteolysis-targeting chimeras (PROTACs) that may open new and innovative ways to benefit from molecular-targeted therapy approaches with improved efficacy.

## Introduction

**Fig. 1:**
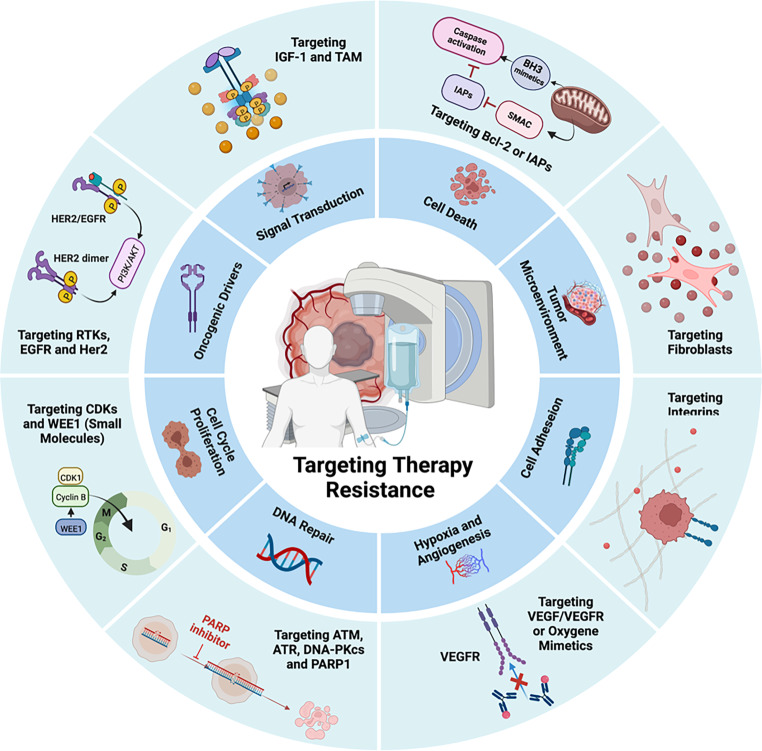
Schematic representation of molecular mechanisms associated with treatment resistance in solid tumors and molecular targeting approaches for cancer chemo- and radiation sensitization. These strategies cover interference with DNA repair, cell cycle progression, oncogenic drivers, cell adhesion, cell death/apoptosis, and signal transduction. In addition, they aim at impacting on tumor hypoxia and angiogenesis as well as the tumor microenvironment. Details are given in the text. *ATM* ataxia telangiectasia mutated kinase; *ATR* ataxia telangiectasia mutated and Rad3-related kinase; *BCL2* B cell lymphoma 2; *CDKs* cyclin-dependent kinases; *DNA-PKcs* DNA-dependent protein kinase, catalytic subunit; *EGFR* epidermal growth factor receptor; *IAP* inhibitor of apoptosis protein; *IGF‑1* insulin-like growth factor‑1; *PARP1* poly(ADP-ribose)-polymerase 1; *RTKs* receptor tyrosine kinases; *Smac* second mitochondrial activator of caspases; *TAM* Axl and MerTK receptor; *VEGF* vascular endothelial growth factor. Created with Biorender.com

A combination of cytotoxic chemotherapy and radiation therapy (chemoradiotherapy, CRT) is still the standard treatment regimen for patients with solid tumors of advanced or metastatic stage. The primary objectives of radiation treatment are to achieve local and regional tumor control and to minimize normal tissue toxicities, referred to as the therapeutic ratio. The efficacy of such current therapeutic approaches, however, is compromised by an inherent or acquired radiation/drug resistance and off-target effects, and not all patients respond to therapy in an adequate manner. Recent progress in understanding the tumor’s biology and cellular response to ionizing radiation (IR), however, have resulted in the identification of an increased number of molecular targets. Comprehensive selection of druggable targets and a deep understanding of tumor radiobiology are prerequisites for successful implementation of personalized medicine approaches in radiation oncology, with the goal of treating individual tumors according to their specific radiobiological vulnerabilities [1]. Exposure of cells to IR induces an intracellular signaling that is multifaceted and involves DNA damage response (DDR), modulation of the cell cycle, and, among other things, execution of cell death by apoptosis or induction of senescence. These cellular events are further affected by a multitude of extrinsic factors including growth factor/cytokine signaling cascades and their underlying kinases as well as oncogenic driver(s) [2]. In addition, the tumor microenvironment, e.g., angiogenesis, levels of hypoxia, and an extensive interrelationship with cellular components of the immune system, stromal fibroblasts, or endothelial cells directly impact on the IR response [3–5]. Over the past decades, these radiation-induced/mediated signaling pathways have been explored in depth for radiation sensitization, resulting in the development of innovative molecular therapies. The principal molecular mechanisms associated with RT resistance in solid tumors and molecular targeting approaches for (chemo) radiosensitization are summarized in Fig. 1. By applying a multitude of pharmacological, genetic, and immunological principles (e.g., small molecules, antibodies, immunotherapy, and RNA-based approaches), targeted strategies were developed that are suitable for multimodal concepts with RT or CRT. Based on a modification of the Steel hypothesis, Bentzen et al. [6] have outlined five mechanisms for how IR and targeted therapy may interact. These mechanisms cover 1) spatial cooperation, 2) temporal modulation, 3) biological cooperation, 4) cytotoxic enhancement, and 5) non-cancerous tissue protection. In addition, by the application of nanoparticular carrier systems, increased and more specific tumor delivery of targeted therapeutics may be achieved [7].

Despite a plethora of promising experimental and preclinical data, however, so far little improvement has been reported in a clinical setting when RT or CRT are combined with targeted therapy and most of studies have failed to show a patient benefit. As a result, only one molecular therapy has so far been approved for use with RT, namely the anti-epidermal growth factor receptor (EGFR) antibody cetuximab in head and neck squamous cell carcinoma (HNSCC) [8, 9]. But even if cetuximab can increase overall survival compared to RT alone [10], cetuximab is clearly inferior to cisplatin-based CRT [11].

The current review article aims to summarize recent progress in molecular targeted therapies combined with radiotherapy (RT) or CRT. Due to the high complexity of this topic, we focus on a clinical evaluation of selected targeting agents but do not include radioimmunotherapy approaches. The review will further summarize and evaluate promising innovative treatment options like RNA technology, protein-degrading proteolysis-targeting chimeras (PROTACs), and nanoparticular platforms to improve on-site effects and to increase the efficacy of future targeted methodologies.

## Targeting DNA damage response for chemoradiation sensitization

CRT induces a multitude of DNA damages which trigger activation of complex DNA damage response (DDR) signaling networks. The principal aim of the DDR is to identify the DNA damage and induce chromatin alterations around it to foster the recruitment of DNA damage- and repair-sensing proteins. This is followed by triggering cell cycle arrest, enabling DNA repair prior to DNA replication or mitosis. A complete overview of the DDR signaling network is beyond the scope of this article but has recently been extensively reviewed in [12, 13]. In brief, for sensing of the DNA double-strand breaks (DNA DSBs) the MRN complex consisting of RAD50, the meiotic recombination 11 homolog 1 (MRE11), and the Nijmegen breakage syndrome protein 1 (NBS1) is critical for the recruitment and autophosphorylation of ataxia telangiectasia mutated (ATM) kinase, thereby triggering several different DNA repair circuits as well as downstream effectors [14, 15]. Beyond sensing of DNA DSBs inflicted by IR, ATM can also be activated in response to stalled replication forks during intrinsic duplication of the genome [13]. The downstream substrates of the ATM kinase include, amongst others, histone H2AX, which, when phosphorylated, forms a scaffold for DDR proteins, the checkpoint kinase 2 (CHK2), and the tumor suppressor protein p53 (TP53) acting on the G1/S passage [13]. Activation of CHK2 and p53 in turn results in cell cycle arrest, permitting DNA repair to take place before the onset of DNA replication, but may also induce cell death or senescence. Another critical DNA damage sensor is the ataxia telangiectasia mutated and Rad3-related (ATR) kinase [16, 17], which is primarily activated through stretches of single-stranded DNA and plays a central role in the repair of DNA strand breaks formed during replication and induction of G2/M cell cycle arrest. Of note, while the initial DNA lesions sensed by ATM or ATR are diverse, downstream signaling cascades are to some extent overlapping and, hence, the integration of ATM and ATR as well as the cell cycle and tumor cell signaling status will influence the net outcome of the DDR [16, 18]. As DNA DSBs can easily result in loss of genetic information and subsequent cell inactivation, their repair is of outmost importance for cell survival and two principal different pathways are involved, i.e., the classical non-homologous end-joining (c-NHEJ) and the homologous recombination repair (HRR) pathway, with the latter requiring an undamaged sister chromatid as an error-free repair template DNA strand copy [19, 20]. In addition, alternative NHEJ and single-strand annealing may also work on the DNA DSB repair in cases where c‑NHEJ and HRR attempts failed; however, these pathways have low repair accuracy and their usage always results in sequence deletions [17].

The DDR sensor and repair proteins, ATM, and ATR, as well as the NHEJ kinase DNA-dependent protein kinase, catalytic subunit (DNA-PKcs), have all been exploited for development of DDR inhibitors. In general, DDR inhibitors use different mechanisms for tumor-selective RT or CRT sensitization. First, they may interfere with DNA repair efficacy or cell cycle checkpoints, resulting in higher residual DNA damage levels or premature passage through mitosis, which damages chromosomes. Secondly, DDR inhibition can result in sensitization by exploiting synthetic lethality, as shown in the context of poly(ADP-ribose)-polymerase (PARP) inhibition in tumors deficient in HRR as well as by ATR blockade in replication-stress-adapted tumors, and, thirdly, by causing a higher tumor mutation rate as a failure of DNA damage repair results in elevated neoantigen expression in line with increased cytotoxic T lymphocyte activity [18, 21].

The rationale for targeting ATR in cancer is that malignant cells are often adapted to replication stress, which requires a functional ATR/CHK1/WEE1 axis, which is particularly evident when certain DNA-damaging agents are used [18]. So far, three ATR inhibitors have reached clinical testing, i.e., berzosertib (M6620 or VX-970), ceralasertib (also known as AZD6738), and elimusertib (BAY1895344) [16, 18, 22, 23]. These ATR inhibitors have been studied in phase I or phase II clinical trials in a monotherapy setting, combined with different chemotherapies, other DNA repair inhibitors such as the PARP inhibitor olaparib, or with immune checkpoint inhibitors [16, 18, 22, 23]. Based on preclinical findings where ATR inhibition in combination with a topoisomerase 1 (TOP1) inhibitor demonstrated synthetic lethality, berzosertib and topotecan, a selective TOP1 inhibitor, were evaluated in different solid tumor malignancies [24] and in a proof-of-concept study in patients with small cell lung cancer (SCLC) [25]. About 36% of these SCLC patients showed an objective response even when having platinum-resistant disease. Berzosertib and ceralasertib have further been combined with gemcitabine or carboplatin in different solid tumors in early-phase trials with some efficacy (NCT02595892, [26–28]). The ATR inhibitor ceralasertib (AZD6738), an oral inhibitor, has also been evaluated in combination with carboplatin in a diverse set of solid tumors [28] (NCT02264678, *n* = 35 patients). About half of the patients had stable disease according to the Response Evaluation Criteria in Solid Tumors (RECIST). Notably, fewer attempts have so far been made with ATR inhibitors in combination with RT (Table 1; [17]). Examples are PATRIOT and CHARIOT. PATRIOT evaluates ceralasertib combined with palliative RT in solid tumors ([29]; NCT02223923), whereas CHARIOT investigates berzosertib in esophageal cancer in combination with RT in the palliative and later on with CRT in the curative setting (NCT03641547). In addition, the ATR inhibitor elimusertib is currently being evaluated in combination with stereotactic body radiotherapy (SBRT) alongside the immune checkpoint inhibitor pembrolizumab in HNSCC (NCT04576091). So far, results of these trials have not been reported.

**Table 1:** Recent molecular sensitizing strategies and exemplary drugs that are in clinical evaluation in combination with (chemo)radiation therapy

Pathway or mechanism	Molecular target	Tumor entity	Inhibitors in clinical use	References/clinical trial registration^a^
DNA damage and repair	ATM	Glioblastoma, other brain tumors, soft tissue sarcoma, non-small cell lung cancer	AZD1390, M3541	[37] NCT03423628; NCT05116254; NCT04550104; NCT03225105
	ATR	Advanced solid tumors, esophageal cancer, head and neck cancer	Berzosertib (M6620 or VX-970), ceralasertib (AZD6738), elimusertib (BAY1895344)	[29] NCT02223923; NCT03641547; NCT04576091
	DNA-PKcs	Advanced tumors, head and neck cancer, solid tumors, soft tissue sarcoma, rectal cancer, prostate cancer	Nedisertib, peposertib, M3814, AZD7648	[41] NCT02316197; NCT05116254; NCT04071236; NCT04750954; NCT03770689; NCT02516813
	PARP	Breast cancer, prostate cancer, non-small cell, small cell lung cancer, glioblastoma, rectal cancer, cervical cancer, head and neck cancer	Olaparib, veliparib, rucaparib, niraparib	[49, 175, 176] NCT04837209; NCT01477489; NCT02227082; NCT03945721; NCT03598257; NCT03109080; NCT03212742; NCT03581292; NCT01514201; NCT04790955; NCT04728230; NCT02412371: NCT01589419; NCT03644342; NCT02229656
Chromatin remodeling	HDAC	Head and neck cancer, pancreatic cancer, brain tumors	Vorinostat	[54] NCT02349867; NCT00983268; NCT01064921; NCT01236560; NCT01189266
Cell cycle progression	WEE1	Pancreatic cancer, diffuse intrinsic pontine glioma	Adavosertib	[33, 34]
	CDK 4/6	Breast cancer, head and neck cancer, diffuse pontine glioma, high grade glioma, meningiomas; prostate cancer	Palbociclib, ribociclib, abemaciclib	[55, 56] NCT03691493; NCT03870919; NCT04563507; NCT03389477; NCT03355794; NCT02607124; NCT04298983
Cell death promotors	BCL‑2	Head and neck cancer, esophageal cancer, glioblastoma	Gossypol/AT-101, navitoclax, venetoclax	[91, 95] NCT00390403; NCT00561197
	CD95L cell death ligand	Glioblastoma	Asunercept/APG101, CAN008	[100] NCT05447195
	IAPs	Advanced head and neck cancer	Xevinapant (Debio 1143, Debiopharm/Merck KGaA)	[110] NCT04459715; NCT05386550; NCT02022098
Oncogenic drivers	EGFR	Non-small cell lung cancer, head and neck cancer	Erlotinib, gefitinib, osimertinib, cetuximab	[10, 59, 64–68]
Angiogenesis	VEGF, VEGFR2	Glioblastoma, rectal cancer, esophagogastric cancer	Bevacizumab (Avastin, Roche Pharma AG), vandetanib	[111–114]
Hypoxia	Oxygen mimetic	Head and neck cancer	Nimorazole	[116, 117]

*ATM* ataxia telangiectasia mutated kinase, *ATR* ataxia telangiectasia mutated and Rad3-related kinase, *Bcl‑2* B cell lymphoma 2, *CDK 4/6* cyclin-dependent kinase 4 and 6, *DNA-PKcs* DNA-dependent protein kinase, catalytic subunit, *EGFR* epidermal growth factor receptor, *HDAC* histone deacetylase, *IAP* inhibitor of apoptosis protein, *PARP* poly(ADP-ribose)-polymerase 1, *VEGF* vascular endothelial growth factor, *VEGFR* VEGF receptor

^a^The trial numbers refer to their citation on https://clinicaltrials.gov/

The Ser/Thr protein kinase WEE1 regulates the S‑ and G2-phase checkpoints in response to DNA damage by directly phosphorylating and inactivating cyclin-dependent kinases 1/2 (CDK1/2), fostering cell cycle arrest. WEE1 is linked to ATR activity through the ATR target CHK1 and, similar to ATR, counteracts replication stress and protects stalled replication forks [30]. In addition, WEE1 positively regulates HRR via modulation of CDK1 and the breast cancer 2 (BRCA2)–RAD51 interaction [31]. Inhibition of WEE1 results in unscheduled mitotic entry, increased replication stress, nucleotide starvation and loss of genomic integrity [32]. In patients with locally advanced pancreatic cancer, the combination of adavosertib, a first-in-class WEE1 inhibitor, with RT and gemcitabine achieved substantially higher survival rates than previous results using RT and gemcitabine alone, which clearly warrants further clinical evaluation [33, 34]. Adavosertib has also been studied for combination with cranial RT in children with diffuse intrinsic pontine gliomas (DIPG), where the combination was tolerable yet not giving improved OS compared to RT based on historical records [34].

The progress in ATM inhibitor development has been challenging, given the similarity to phosphoinositide 3‑kinases (PI3K) and phosphatidylinositol 3‑kinase-related (PIKK) kinase domains, with most of the developed drugs showing non-specificity [22, 23]. One small molecule is AZD0156, which is currently being studied for tolerability in patients with advanced metastatic tumors and tested in combination with irinotecan or olaparib (NCT02588105), since these combinations were effective in preclinical models [35]. A further development is AZD1390, which sensitizes for RT in preclinical tumor models [36]. As compared to AZD0156, the drug also penetrates through the blood–brain barrier and, hence, may offer a way to treat brain tumors as well as metastatic lesions in the brain. Along this line, AZD1390 combined with partial or whole-brain irradiation is being explored in a phase I trial in patients with glioblastoma as well as in patients with brain metastases (NCT03423628). AZD1390 is also being tested in combination with RT in adult patients with soft tissue sarcomas for RT sensitization purposes and improved local control (NCT05116254), as well as in NSCLC of different stages combined with curative RT in the CONCORDE study (NCT04550104; Table 1). Another ATM inhibitor, M3541, was investigated in patients with malignant lesions in the thorax, abdominal cavity, head and neck region, or extremities (any histology) likely to benefit from palliative RT (NCT03225105). The trial closed early due to the absence of a dose-response relationship and a non-optimal pharmacokinetic profile; the authors did not recommend further clinical development [37].

Although it was realized early on that targeting DNA-PKcs would be an option for radiosensitization given its central role in c‑NHEJ, the similarity of the kinase domain to other PI3K kinases made it challenging to develop specific inhibitors [20, 22]. Recently, however, progress was achieved, with several small molecules being developed to more specifically block DNA-PKcs kinase function. AZD7648 is reported to augment RT response in murine tumors [38, 39] and is now being studied in combination with RT for patients with soft tissue sarcoma (NCT05116254). Nedisertib or peposertib (also referred to as M3814) represent other DNA-PK inhibitors which have been shown to sensitize tumor cells towards RT (reviewed in [13]) and to selectively accumulate in melanoma brain metastases [40]. Peposertib is combined with RT in different advanced tumor malignancies including rectal cancer (NCT03770689, NCT02516813; Table 1). By this, van Bussel et al. recently published the first results of peposertib treatment in patients with advanced solid tumors [41]: 31 patients were included; stable disease was achieved in 12 patients, lasting for more than 12 weeks in 7 patients. Peposertib is currently being further evaluated in combination with different radionuclide therapies. Thus, it is combined with radium-223 dichloride or avelumab in patients with metastatic prostate cancer non-responsive to hormonal therapy with or without immune therapy (NCT04071236), or is added on top of approved lutetium Lu-177 treatment for neuroendocrine tumors (NCT04750954). In summary, the efficacy of these ATM and DNA-PKcs inhibition trials is yet to be reported and it will also be interesting to what extent radiosensitization conferred through these approaches will be tumor specific, since both proteins are also mandatory for effective DNA repair in normal tissue cells and deficiencies result in severe radiosensitivity [42, 43].

A further clinically established subset of DNA repair inhibitors are those aiming to inhibit PARP1 [13, 44]. The development of PARP inhibitors (PARPi) was a breakthrough, exemplifying the concept of “synthetic lethality” [45, 46]. Thus, in cancer cells lacking DNA repair functions because of mutated DDR genes impairing a specific repair pathway, the pharmacological inhibition of back-up or alternative pathways may result in pronounced tumor-specific cell killing [47]. The principal clinical example is BRCA1/2-deficient tumors, which are highly sensitive to PARPi. In addition, synthetic lethality with PARPi has also been shown in tumors with deficiencies in ATM function, such as ovarian, breast, and prostate cancer [48]. In the context of RT, PARPi are currently being explored in several different tumor entities, e.g., breast cancer (NCT04837209; NCT01477489; NCT02227082; NCT03945721; NCT03598257; NCT03109080), glioblastoma/glioma (NCT03212742; NCT03581292; NCT01514201), small cell lung cancer (NCT04790955; NCT04728230), non-small cell lung cancer (NCT02412371), rectal cancer (NCT01589419), and cervical cancer (NCT03644342), as well as in HNSCC (NCT02229656). PARPi are further being intensively evaluated in metastatic castration-resistant prostate cancer ([49]; Table 1). In summary, PARPi is a unique example of where DNA repair inhibitors have shown great promise in a clinical setting with successful transfer of knowledge from the bench to the bedside. Similar to other small molecules, acquired resistance to PARPi is often seen, which impairs a durable response and, accordingly, hampers overall survival in the clinics [50]. In addition, tumor specificity may remain a challenge, because in some entities, the maximum tolerated doses (MTD) of PARP inhibitors with RT and especially CRT can be severely reduced as compared to their use as monotherapy [51, 52].

Another class of inhibitors currently applied in clinical evaluation is tailored to chromatin regulation and includes histone deacetylase (HDAC) inhibitors (HDACi). The state of chromatin condensation can influence the accessibility of DNA repair enzymes to the DNA DSBs, with DNA methylation or histone methylation or acetylation to cover epigenetic modifications that affect DNA repair ability [53]. Indeed, tumor cells of different origins have been shown to be sensitized for RT when HDACi such as vorinostat, valproic acid, trichostatin A, or panabinostat are used, with DDR signaling and other IR-induced signaling events being reported (reviewed in [53]). Several clinical trials are indeed evaluating the HDACi vorinostat in combination with CRT. A phase I trial applying vorinostat in combination of the drug with concurrent CRT has been published to reveal complete response to therapy in 24 out of 26 patients with advanced stage, mostly human papillomavirus (HPV)-positive, HNSCC [54]. Thus, a high rate of complete responses with toxicity rates comparable to standard treatment strongly encourage further clinical assessment in phase II and III trials. Vorinostat is also being explored in combination with CRT alongside sorafenib in pancreatic cancer patients who have been given neoadjuvant chemotherapy (CT; NCT02349867) as well as in non-metastatic pancreatic cancer where it has been tested in combination with capecitabine and RT (NCT00983268). Further, vorinostat is being explored with cisplatin-based CRT in patients with unresectable cancer of the oropharynx (NCT01064921). Moreover, vorinostat is also studied for its capacity to potentiate CRT in younger patients with different brain malignancies, e.g., anaplastic astrocytoma, oligoastrocytoma, gliosarcoma, and pontine glioma, with the secondary objective of evaluating the effect on DDR signaling in PBMC as well as in tumor specimens (NCT01189266). In addition, there is a randomized phase II/III study (NCT01236560) aiming to explore the effect of vorinostat on local irradiation in children with high-grade gliomas, where temozolomide and bevacizumab are used as maintenance regimens (Table 1).

Finally, as cyclin-dependent kinase 4 and 6 inhibitors (CDKIs), such as palbociclib, ribociclib, and abemaciclib, have become a mainstay in the treatment of metastatic breast cancer, they are partly combined with palliative radiotherapy. The toxicity of combined treatment is highly dependent on the location of the metastatic lesions. While the clinical purpose was rather to achieve additive effects of both modalities, mostly in a temporally separated sequence, preclinical as well as some clinical studies have also begun to explore the radiosensitizing potential of these inhibitors ([55–57]; Table 1).

## Targeting oncogenic drivers and signaling pathways

Numerous preclinical and clinical results demonstrate that a constitutively increased activity of ErbB receptors (e.g., EGFR) by mutation (i.e., primarily in non-small cell lung cancer) or gene amplification (in multiple tumor types including head and neck cancer) is responsible for both chemo- and radiation resistance [58, 59]. Since the approval of imatinib, which targets the Abelson (ABL) tyrosine kinase deregulated by gene fusion (BCR-Abl) in almost all cases of chronic myeloid leukemia, about 50 kinase inhibitors [60] and a number of monoclonal antibodies [61] have been clinically approved for cancer treatment. Due to the possibility of inhibiting individual tumors‘ oncogenic drivers, targeting signal transduction pathways represents one of the key strategies of current precision medicine approaches in oncology, although resistance mechanisms, such as activating downstream mutations or the activation of alternative signaling pathways, occur frequently with time.

Regarding the combination with IR, the inhibition of signal transduction pathways has been extensively studied in the preclinical setting and, apart from promoting proliferation and inhibiting apoptosis, these pathways have further been shown to stimulate the repair of radiation-induced DNA damage and contribute to RT resistance. Especially the inhibition of receptor tyrosine kinases (TKI), such as the ErbB family members EGFR and HER2, the insulin-like growth factor (IGF-1) receptor, or the Tyro3, Axl, and MerTK (TAM) receptors, but also components of main downstream signaling pathways, such as the RAS-RAF-MEK-ERK (MAPK), the phosphatidylinositol-3-kinase (PI3K)/Akt, and the mammalian target of rapamycin (mTOR) signaling pathway are well-described approaches for RT sensitization using either small tyrosine kinase inhibitors (TKIs) or blocking antibodies [62, 63]. The combination of the anti-EGFR antibody cetuximab and RT received clinical approval for the treatment of HNSCC in 2008, but in fact, this has since remained the only approved molecular targeting approach in combination with RT. Furthermore, a number of more recent results question the efficacy of this combination [9]. Especially the inferiority of cetuximab and RT to cisplatin-based CRT in HPV-positive oropharyngeal SCC, as observed in three similar phase III trials [64–66], and the lack of benefit when adding cetuximab on top of cisplatin-based CRT [67] call into question whether cetuximab is indeed capable of inducing effective sensitization to RT in HNSCC. Along this line, a recent meta-analysis covering 13 multi-arm randomized controlled trials with 5678 patients on CRT-based treatment and inhibition of the EGFR, HER2, or VEGFR pathway in solid cancers (ROCKIT) concluded that molecular targeting conferred no benefit on survival but instead increased grade 3 toxicity [68]. Similarly, no benefit from adding TKIs to radiation was observed in a meta-analysis of overall 30 smaller studies on brain metastasis from NSCLC with EGFR or ALK mutations [69].

What might be the reasons for the lack of effectiveness in the clinical setting observed over the last decade? In preclinical models, cellular resistance mechanisms against signal transduction pathway inhibition, such as the activation of a compensating pathway or activating mutations in downstream pathway members, have been described to impair the RT capacity. In the in vitro setting, such mechanisms could often be tackled through combined molecular targeting approaches. In contrast, in the clinical setting, data are sparse, and it is currently difficult to estimate to what extent compensating mechanisms contribute to the observed lack of sensitization and whether combined targeting strategies will be beneficial [70]. Another highly challenging obstacle is the lack of predictive biomarkers (BMs). Also, for the approved agent cetuximab, EGFR receptor mutations or the expression level have not been shown to influence efficacy [10]. Similarly, no predictive BMs have been established for the inhibition of other signal transduction pathways when combined with RT. Without specific BMs, potentially responding subpopulations could remain undiscovered in unselected, larger, and overall poorly responding study populations. An attempt to overcome this issue is the NCT Neuro Master Match (N2M2) trial, a multicenter phase I/IIa umbrella trial for isocitrate dehydrogenase (IDH) wildtype, unmethylated glioblastoma. Within 4 weeks after surgery, tumors are molecularly characterized and, in case of targetable alterations, patients are stratified for RT with either alectinib, idasanutlin, palbociclib, vismodegib, or temsirolimus (inhibiting ALK-fusions/point mutated ALK, the p53-MDM2 interaction, CDK4/6, hedgehog, or mTOR, respectively). Progression-free survival (PFS) after 6 months will serve as the primary endpoint in the phase II part of the trial [71].

In the metastatic situation, promising results were reported for the combination of the EGFR/HER2 inhibitor lapatinib and RT for brain metastases derived from HER2-positive breast cancer. Several retrospective studies further point towards enhanced survival and, especially for the use of stereotactic radiosurgery (SRS), also towards enhanced local control without enhanced rates of radionecrosis [72, 73]. As both sequential and concomitant administration of lapatinib conferred a survival benefit, it is currently unclear to what degree synergistic mechanisms at the cellular level are involved and to what extent a disruption of the brain–blood barrier through RT may have improved the access of lapatinib. Despite open questions regarding the underlying mechanisms, a prospective evaluation of the efficacy of the approach appears warranted.

Pending results of a profound number of ongoing clinical trials, it still remains to be shown whether molecular targeting of signal transduction pathways that drive oncogenic transformation might not only reduce tumor growth and prolong survival, but also improve the efficacy of RT to ultimately increase cure rates in the curative setting.

## Targeting cell adhesion molecules to booster the radiation response

Cell–matrix interactions are mainly mediated by a family of transmembrane cell adhesion molecules named integrins. Beyond physiological cell adhesion and migration processes, they also contribute to cancer progression, invasion, and metastatic spread, but also confer radio- and chemoresistance of tumor cells through the activation of signaling pathways, often in concert with growth factor receptor signaling [74–76]. As part of multiprotein focal adhesion complexes, integrins can be assembled into 24 presently known heterodimer combinations of 18 α- and 8 β-integrin subunits to allow for differential recognition of distinct environments [77]. A well-studied subfamily including the heterodimeric cell surface receptors αvβ1, αvβ3, αvβ5, and α5β1 integrins bind to an Arg-Gly-Asp (RGD) motif present in extracellular matrix proteins such as fibronectin, fibrinogen, vitronectin, or osteopontin. Upon binding to extracellular matrix proteins, integrins recruit intracellular signaling molecules, with focal adhesion kinase (FAK) being one of the main effectors of integrin signaling. Overexpression of FAK is reported in a huge number of different cancers, where it controls migration and pro-survival signaling upon stress (reviewed in [78, 79]). Regarding responsiveness, especially β1 integrins are well described to confer radio- and chemoresistance to tumor cells through the activation of FAK and signal transduction pathways, often common with those stimulated by growth factor receptors. For example, there is evidence that single inhibition or knockdown of β1 integrin in glioma cells [80] or of FAK in HNSCC [81] or ductal carcinoma in situ (DCIS) of the breast [82] results in radiosensitization. Dual targeting of EGFR and β1-integrin by inhibitory antibodies cetuximab and AIIB2, respectively [76], or EGFR and FAK [83], further enhanced the radiation response of HNSCC when compared with single treatment.

The clinically most advanced strategy of integrin targeting in combination with RT is selective inhibition of αvβ3 and αvβ5 integrins by cilengitide. The approach was less based on inhibition of cellular radiation resistance mechanisms, but was rather intended to add the inhibition of invasion, tumorigenesis, and angiogenesis to standard-of-care RT regimes [84]. Unfortunately, despite promising data from phase II studies [85–87], cilengitide did not improve patient survival in a multicenter, randomized, open-label phase III trial in newly diagnosed O(6)-methylguanine-DNA-methyltransferase (MGMT) promoter methylated glioblastoma when added to temozolomide-based CRT (CENTRIC EORTC 26071-22072 study, NCT00689221) [88]. Nevertheless, analysis of the levels of αvβ3 and αvβ5 integrins, the targets of cilengitide, in tumor samples from glioblastoma patients enrolled in the phase II CORE clinical trial revealed that higher αvβ3 levels in tumor tissue were associated with improved PFS and OS in patients lacking MGMT promoter methylation treated with cilengitide. Another compound, GLPG0187, a broad-spectrum integrin antagonist, was tested in a dose-escalating phase I study in patients with progressive glioma and advanced solid malignancies, but failed to show effectiveness as monotherapy [89].

The central role of FAK in mediating integrin signals and its common overexpression in cancer has prompted the development of a variety of kinase inhibitors which were also tested in clinical trials but with poor results when applied as monotherapy (reviewed in [78]). As mentioned above, targeting FAK in combination with additional inhibitors and/or RT/CRT might be more effective to overcome radio- and/or chemoresistance. Accordingly, it will be interesting to see the results of a current phase II study applying the FAK kinase inhibitor defactinib (VS-6063, PF-04554878) in combination with CT followed by SBRT in advanced pancreatic adenocarcinoma (NCT04331041).

Despite the so far failed implementation of adhesion molecule targeting in the clinical setting, possibly due to a lack of BM signatures to select for eligible patients and restricted early-phase clinical trials to carefully unravel dose- and time-dependent administration of substances, preclinical data suggest a great potential for the integration of ideally multiple compounds to circumvent resistance mechanisms in combined therapies [84].

## Interfering with apoptosis signaling for chemoradiation sensitization

Resistance to cell death by apoptosis is a pivotal mechanism in the molecular pathogenesis and in treatment resistance in cancer cells [2]. Current molecular strategies to increase tumor response thus aim at restoring the apoptosis sensitivity with rebuilding of apoptotic signaling via the mitochondrial route by targeting B‑cell lymphoma 2 (BCL-2) family proteins [90–92]. Thus, synthetic molecules have been developed that are mimetics of the BH3 domain-only proapoptotic BCL‑2 protein or which target different proapoptotic BCL‑2 family members like BAX and BAK to allow for release of apoptogenic factors from the mitochondria resulting in downstream activation of caspases. These BH3 mimetic drugs were shown to potentiate cell death in different solid tumor cells in vitro as monotherapy or combined with RT or CT. Some examples of these agents cover gossypol (AT-101) and navitoclax (ABT-263), which act on BCL‑2, BCL‑X, and BCL‑W, and more specific agents such as venetoclax (ABT-199) restricted to BCL‑2 [90, 91]. While gossypol and its more stable enantiomer AT-101 have been stated to be tolerable when used in mono- or combined modality treatment [91], navitoclax/ABT-263 was shown to have an effect on platelet count, while venetoclax/ABT-199 caused neutropenia and gastrointestinal adverse effects [92, 93]. Gossypol and AT-101 were primarily evaluated in a regimen with CT in patients with different solid malignancies but also with CRT in some patient subsets [91]. In vitro studies in HNSCC cell lines showed that AT-101 potentiated RT, decreased viability, and increased caspase-mediated apoptosis [94]. Of note, these effects were evident when applying AT-101 at a concentration achievable in plasma of HNSCC patients (10–20 mg in different scheduling). A phase I/II trial evaluated AT-101 combined with preoperative CRT (docetaxel, fluorouracil) in esophageal cancer (EC) patients (NCT00561197) [95]. Although some patients experienced toxicity, most of the patients had a clinical complete response evident as tumor-negative biopsies or lack of positron-emission tomography uptake in the former tumor lesions. When posttreatment biopsies where studied, reduced cancer stem cell (CSC) marker expression, i.e., YAP and SOX9, was evident, suggesting that in vivo, AT-101 may, apart from acting on BCL‑2 expression, also be able to target CSC, a fraction of cells often refractory to RT. AT-101 was further studied in glioblastoma multiforme either alone (NCT00540722) or combined with temozolomide and RT (NCT00390403; Table 1). Both studies have completed recruitment, yet publication of the effects of combining AT-101 with CRT is pending.

Concerning other substances, published data are restricted to in vitro investigations at present. Navitoclax (ABT-263) is reported to potentiate CT and/or RT in multiple cancer cell lines including HPV-negative HNSCC cells, resulting in synergistic effects with reduced cell viability and increased cell death in part via apoptosis [96–98]. There have also been attempts to restore the function of pro-apoptotic BCL‑2 family members such as BAK. Thus, it was demonstrated that BKA-073, a small molecule inhibitor capable of turning BAK into an active form allowing mitochondria-mediated apoptosis to proceed, could trigger apoptotic cell death in NSCLC and SCLC cells as well as in patient-derived xenografts in vivo [99]. It will be interesting to see if such an approach can also sensitize for RT in a clinical setting.

Another way to improve cell death induction is to target cell death receptor ligands such as FASL/CD95L with asunercept (APG101), which is a fusion protein targeting the CD95 ligand (CD95L). In a phase II study (NCT01071837), glioblastoma patients were randomized to receive either fractionated second RT (total dose 36 Gy) alone or combined with APG101 [100]. Results indicated PFS rates at 6 months of 3.8% for patients receiving RT, while the rate was 20.7% for patients receiving RT combined with APG101, and a median PFS of 2.5% and 4.5%, respectively. Following up on the mechanisms behind, Blaes et al. demonstrated that APG101 blocked glioma cell invasion in vivo and APG101 treatment in combination with RT resulted in reduced tumor volumes and increased survival in a glioma mouse model [101]. In an ongoing multicenter phase II study (NCT05447195), APG101, also known as CAN008, is being evaluated combined with temozolomide-based CRT after tumor resection followed by adjuvant temozolomide.

Overexpression of inhibitor of apoptosis proteins (IAPs), which can impede caspase activation but also affect malignant cell survival in other ways [102], is another apoptosis resistance mechanism of cancer cells. Irregularities in the activation of IAPs, well studied on the smallest member of the family, survivin, is linked to disease onset, progression, and treatment resistance of various malignancies [103]. Survivin has indeed been in focus as a cancer therapy target, as it plays a central role in multiple cancer hallmarks including proliferation, metastasis, and DNA repair. Thus, multiple ways to target survivin have been developed within recent decades [104, 105]. Survivin-directed therapy has also been evaluated in phase I/II trials of leukemia, NSCLC, and prostate cancer, using either antisense oligonucleotide LY2181308 (gataparsen) or YM155, which is an inhibitory imidazolium-based small molecule. The results obtained when used in monotherapy were rather modest in NSCLC, while in prostate cancer, the treatment caused disease progression [106, 107]. Second mitochondrial activator of caspases (Smac) is a mitochondrial protein which prohibits IAPs function [108]. Accordingly, mimetics of Smac were developed which, in vitro, potentiated both CT and RT effectiveness in various cancer cell line models [108]. Xevinapant (Debio 1143, Debiopharm/Merck KGaA) is an orally applicable Smac mimetic which has been shown to enhance RT in HNSCC cell lines in vitro and to sensitize HNSCC xenografts for RT in vivo via apoptosis induction and increased tumor necrosis factor alpha (TNFα) expression [109]. Recently, xevinapant has been evaluated in combination with high-dose cisplatin CRT in locally advanced HNSCC patients by the French Head and Neck Radiotherapy Oncology Group (GORTEC) [110] (NCT02022098). In total, 96 patients were randomized 1:1 to receive oral Debio 1143 and standard CRT or oral placebo and CRT respectively. Locoregional control 18 months after CRT was observed in 26 out of 48 patients when adding Debio 1143, while the corresponding values for patients given oral placebo was 16 out of 48 patients. However, treatment combination with Debio 1143 also caused slightly higher toxicity, while serious treatment adverse events were similar in both treatment arms. In conclusion, these results strongly suggest that adding the IAP inhibitor to high-dose cisplatin CRT improved outcome, calling for further phase III studies. In line with that, the United States Food and Drug Administration (FDA) also gave breakthrough therapy designation for Debio 1143 combined with standard treatment for HNSCC patients. Several trials with Debio 1143 and different RT regimens are indeed ongoing in HNSCC (NCT04459715; NCT05386550; Table 1) as well as in combination with immune checkpoint inhibitors in different tumor entities (NCT04122625; NCT03270176; NCT03871959), further illustrating the interest in this drug and high clinical potential in this CRT-sensitizing strategy.

## Targeting hypoxia and the tumor microenvironment to impact treatment resistance

Besides tumor cell intrinsic factors, exogenous and tumor microenvironment conditions including tumor vasculature, hypoxia, and stromal components impact on the cellular radiation response [3] and thus constitute valuable targets for radiation sensitization. The influence of the microenvironment for tumor development, progression, and resistance to treatment is well established (reviewed in more detail in [3, 4]). Over the years, for instance, different strategies have been tested to attack hypoxia, which causes RT resistance, primarily through a reduced induction of IR-induced DNA damage. These approaches include drugs that are activated by hypoxia and small molecules that are specifically active in hypoxic cancer cells. Other research lines aim at normalizing the angiogenesis/vasculature often deregulated in tumors by blocking vascular endothelial growth factor (VEGF) or its associated receptors 1/2 (VEGFR1/2).

Regarding VEGF inhibition, although conflicting findings exist, a recent meta-analysis indicated that addition of the anti-VEGF monoclonal antibody bevacizumab (Avastin, Roche Pharma AG) to temozolomide-based CRT in patients with glioblastoma multiforme resulted in enhanced/improved PFS [111], but inconsistent results on OS must be considered [112]. In contrast, in rectal cancer, recent results did not demonstrate that addition of bevacizumab to capecitabine and RT resulted in significant advantages in terms of pathological complete response and 5‑year disease-free and overall survival [113]. Furthermore, vandetanib (ZD6474), a TKI mainly acting on the VEGFRs but also EGFR and the rearranged during transfection (RET) tyrosine kinase, was reported to induce a pathologic complete response and OS of 3.2 years in 5 out of 9 patients with locally advanced esophagogastric carcinoma following paclitaxel, carboplatin, 5‑fluorouracil, and RT [114].

The clinically best-established approach for radiosensitization by targeting hypoxia is the addition of nimorazole, an oxygen mimetic which possesses affinity for electrons [115, 116]. It is long since established that nimorazole specifically targets hypoxic tumor cells to cause RT sensitization, and already in 1998 was it demonstrated to enhance locoregional control by 16% when used in combination with RT as compared to RT alone in a phase III trial in HNSCC [117]. In fact, nimorazole is clinically applied as a standard-of-care RT sensitizer in HNSCC, but only in Denmark [118]. Regarding the combination with CRT, a feasibility phase I/II trial on 50 patients with locally advanced HPV/p16-negative HNSCC treated with hypofractionated accelerated RT in conjunction with cisplatin and nimorazole demonstrated acute toxicity in a rather large proportion of patients (67% severe dysphagia and 61% severe mucositis), but the patients had manageable late toxicity, and locoregional failure and OS at 3 years were promising [116].

Signaling in the tumor microenvironment, including the activity of cancer-associated fibroblasts (CAFs), has recently been demonstrated to be linked to a poor therapeutic response to neoadjuvant CRT in rectal cancer patients with advanced disease. More specifically, upon radiation treatment, interleukin-1α (IL-1α) secretion from cancer cells not only polarizes CAFs toward an inflammatory phenotype (iCAFs), but also triggers oxidative DNA damage, thereby predisposing iCAFs to p53-mediated treatment-induced senescence and extracellular matrix (ECM) accumulation, with the latter resulting in CRT resistance and disease progression [5]. Further, by applying the IL‑1 receptor antagonist (IL‑1 RA) anakinra, these mechanisms could be reverted in vitro and in a mouse model, indicating the drug to constitute a valuable therapeutic option. Indeed, a phase I trial (ACO/ARO/AIO-21 study) was recently initiated to explore the potential of IL‑1 inhibition to overcome treatment resistance and improve response rates to capecitabine-based CRT in rectal cancer [119].

While the previous sections have focused on molecular targeting approaches for sensitization towards RT and CRT that are already being clinically explored, the next sections will provide an overview of novel concepts and technologies that are emerging to have a meaningful impact on future cancer treatment and, in the long run, also hold promise for combined treatment approaches to increase RT/CRT effectiveness.

## Therapeutic targeting of noncoding RNAs (ncRNAs): RNA technology

Besides being established in transferring genetic information into protein sequences, ribonucleic acid (RNA) has critical functions in the regulation of gene expression, impacting on a variety of cellular pathways. In line with this, a multitude of genes in the human genome generate RNA transcripts, called non-coding RNA (ncRNA) that do not translate into proteins but display regulatory properties [120, 121]. Further, RNA species, based on their unique complexity and pronounced diversity, impact on multiple cancer circuits such as proliferation, apoptosis, invasion, metastasis, and genomic stability, thus rendering them valuable targets for developing molecular targeted strategies [122].

Classifying ncRNAs is a matter of constant change. A recent classification covers, amongst other things, the major ncRNA subclasses: microRNAs (miRNAs), long non-coding RNAs (lncRNAs), short non-coding (sncRNAs), P‑element-induced wimpy testis (PIWI)-interacting RNAs (piRNAs), and circular RNAs (circRNAs). Among the most studied ncRNA species, miRNAs encompass a class of endogenous 17- to 25-nucleotide single-stranded RNAs that function as both oncogenic (oncomiRs) or oncosuppressor miRs [123, 124]. In the nucleus, primary miRNAs transcripts (pri-miRNAs) are processed by the ribonuclease Drosha and microprocessor complex subunit Dgcr8 to result in precursor miRNAs (pre-miRNA) of 70 nucleotides in length. Following nuclear export mediated by the protein exportin 5 and Ran-GTPase, pre-miRNAs are next cleaved by the RNase III enzyme Dicer to yield a mature, double-stranded miRNA/miRNA complex. Finally, by incorporation of the miRNA guide strand into the RNA-induced silencing complex (RISC) composed of proteins of the Argonaute family, gene regulation is mediated by degradation of target messenger RNAs [125].

The most abundant class of cellular non-coding transcripts are the lncRNAs, which cover transcripts of 200 or more nucleotides. They exhibit a higher tissue specificity compared to protein-coding transcripts and are reported, amongst other functions, to be involved in transcriptional regulation and alternative splicing [120, 126]. The piRNAs are ncRNA molecules of around 24–31 nucleotides comprising regulatory roles for the PIWI protein family, which cover an additional subclass of RISC constitutes [120, 127]. Functionally, piRNAs act on downstream target genes by repressing transposable elements’ mobilization, thus preserving genomic integrity. Finally, circRNAs are characterized by their specific structure with covalently closed loops, where the 3′ and 5′ ends are joined. Due to their unique circular structure, circRNAs are reported to exhibit higher stability in comparison to other RNA species [128].

**Fig. 2:**
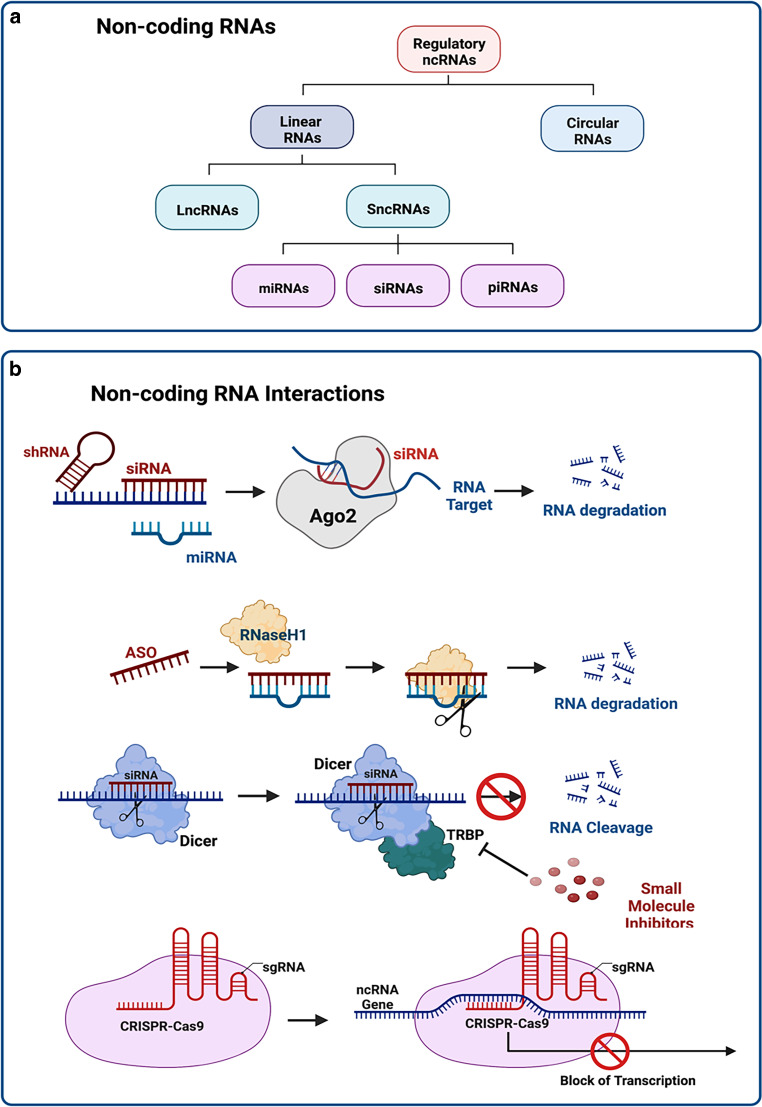
**a** Simplified schematic overview on non-coding RNA (*ncRNA*) species with relevance for molecular targeted strategies in future radiation oncology. According to their structure, these RNA species can be divided into circular and linear RNAs, with the latter to include long non-coding RNAs (*lncRNAs*) and short non-coding RNAs (*sncRNAs*) that, in turn, cover microRNAs (*miRNAs*), small interfering RNAs (*siRNAs*), and P‑element-induced wimpy testis (PIWI)-interacting RNAs (*piRNAs*). **b** Examples of molecular targeting using RNA-based technologies. MicroRNA (*miRNA*) mimics and artificially synthesized small interfering RNAs (siRNAs) or more stable short hairpin RNAs (*shRNAs*) cover endogenous or exogenous double-stranded RNAs which bind to complementary target sequences once bound to the Argonaute RISC catalytic component 2 (*Ago2*), finally resulting in the degradation of the target RNA. Antisense oligonucleotides (*ASOs*) allow for sequence complementary RNA degradation by RNase‑H-mediated cleavage. Small molecule inhibitors act by interfering with RNA transcription in different steps, e.g., processing by Dicer nuclease, while CRISPR/Cas9-mediated gene editing approaches precisely cut the desired target sequence as a consequence of delivery of a Cas9 nuclease in complex with the synthetic guide RNA (*gRNA*). Created with Biorender.com

The discovery of RNA-based gene silencing technologies in the late nineties was a milestone in the field of RNA biology, with a seminal paper published by Fire and Mello to first describe the impact of double-stranded RNAs in post-transcriptional gene silencing with the latter mechanism to cover RNA interference (RNAi) [129]. Within the past two decades, multiple RNA-based therapies and ncRNA inhibitors, including antisense oligonucleotides (ASOs), small interfering RNAs (siRNAs), short hairpin RNAs (shRNAs), anti-miRNAs (anti-miRs), double-stranded miRNA mimics, miRNA sponges, therapeutic circRNAs, clustered regularly interspaced short palindromic repeats/CRISPR associated protein 9, (CRISPR/Cas9)-mediated genome editing, and small molecule inhibitors of ncRNAs have been developed [121, 130–133]. A simplified and exemplary overview of ncRNA therapeutic approaches currently in use or planned for clinical use is depicted in Fig. 2.

ASOs, for instance, cover single-stranded DNA constructs that bind to complementary target RNA sequences to mediate their degradation via cleavage by RNase‑H [134]. The shRNAs were developed to cope with the short lifespan of synthetic siRNAs and are accordingly used as a regular RNAi approach in gene therapy and in clinic [135]. By this, within recent decades, multiple efforts have been made towards a clinical application of RNA-based therapeutics, employing mainly ASOs and siRNAs/shRNAs. At present, more than ten therapeutics have received approval from the FDA or the European Medicines Agency (EMA) aimed at gene therapy in liver, muscle, or the central nervous system. However, results reported so far are ambivalent, with some studies indicating compelling efficacy and others demonstrating a limited effect or toxicity (reviewed in [121]).

The use of RNA-based therapeutics in clinical routine, however, is still hampered by some critical challenges that include 1) on-target effects mediated by uptake in cells other than the target cells, 2) off-target effects based on target sequence similarities or overdosing, 3) vulnerability to serum nucleases, 4) stimulation of an immune response, and 5) oversaturation of the RISC complex [136]. The major obstacle, RNase sensitivity, for instance, can be affected by chemical modifications including substitution with fluorine (2′-F), O‑methyl (2-O-Me) or amine (2′-NH2) to result in degradation-resistant species with proper biological functions [137]. In addition, alternative hydrogen bonding patterns, including wobble and Hoogsteen base pairs, favor an enhanced RNA duplex stability [138]. Moreover, for efficient in vivo delivery, viral vector systems and non-viral delivery systems have been developed that include lentiviruses, adenoviruses, and adeno-associated viruses capable of efficiently delivering vectors encoding miRNA mimics and anti-miRNAs into nuclei [139]. In addition, a genetically engineered bacterium, the EnGeneIC delivery vehicle (EDV) nanocell platform, was established to deliver various therapeutic agents [140]. Notably, EDV nanocells charged with an miR-16 mimic and targeted to EGFR-expressing cancer cells with a bispecific antibody have entered a phase I clinical trial (NCT02369198) in patients with recurrent mesothelioma and NSCLC.

With progress in molecular biology and biotechnology, functional studies of ncRNAs may offer innovative perspectives on cancer treatment. In line with that, some ncRNAs have been proven to be associated with tumor stage, prognosis, and treatment response. Further, ncRNAs have been reported to modulate IR response by impacting on signaling pathways, including DDR, apoptosis, glycolysis, cell cycle, and autophagy [121, 126]. Among the ncRNAs implicated in radiation response, linc00312 was shown to be elevated in patients with nasopharyngeal cancer and complete treatment response as compared to patients with partial response or progressive disease [141]. At a functional level, linc00312 binds the catalytic subunit of DNA-PKcs, thus inhibiting its interaction with the Ku80 subunit following DNA DSB induction [141]. Another example is miR-193b in patients with esophageal cancer treated with concurrent CRT, with only 30–50% of the patients achieving a stable response. Radiation resistance has been connected to the upregulation of miR-193b, with serum levels of the latter found to be significantly lower in patients who exhibited a complete response [142]. Finally, there is accumulating evidence that the ATM kinase controls miRNA expression, for instance, of miR-205-5p [143], by multiple steps, including transcription, maturation, nuclear export of pre-miRNAs, and activation in response to ionizing radiation (for a review see [144]). In addition, several miRNAs were identified that bind to the 3′-untranslated region of ATM to repress its expression or function. Prominent examples of these miRNAs are miR-101 that, upon binding to ATM and DNA-PKcs, diminishes both HRR and NHEJ activity [145], and miR181, which negatively correlates with ATM expression [146]. Finally, van Roosbroeck et al. recently demonstrated that oncomiR miR-155 is implicated in resistance to multiple chemotherapeutic drugs by acting in a reciprocal double-negative feedback loop with p53 [147]. In addition, poly lactic-co-glycolic acid nanoparticles loaded with anti-miR-155 demonstrated reversal of tumor chemoresistance without induction of toxicity [147]. Consequently, based on encouraging findings in in vitro models, anti-miR-155 (MRG-106) is now being evaluated in a phase II study in patients suffering from cutaneous T‑cell lymphoma and mycosis fungoides (NCT03837457).

## Proteolysis-targeting chimeras (PROTACs): a novel class of therapeutics

Besides classical strategies attacking marker mRNA expression and functions as reviewed above, targeted protein degradation has recently emerged as a novel topic in cancer treatment strategies, with proteolysis-targeting chimeras (PROTACs) being most explored. PROTACs are now recognized as a groundbreaking concept in drug discovery with the potential to deliver an entirely new type of small molecule drug that selectively degrades cancer-mediating targets [148–150]. The PROTAC technology takes advantage of the cellular protein degradation machinery, namely the ubiquitin–proteasome system (UPS), to selectively target and degrade specific proteins. PROTACs are heterobifunctional molecules covering an E3 ligase ligand which is linked to a protein of interest (POI) ligand. In cells, PROTACs induce complexes between E3 ligase and the POI, subsequently resulting in polyubiquitination and finally proteasome-mediated degradation of the target protein [148, 151]. In contrast to “conventional” targeted therapies employing small molecules to inhibit enzymatic function by competitive inhibition of druggable active sites, this technology degrades the POI, abolishing not only its enzymatic activity but also possibly circumventing some protein-intrinsic resistance mechanisms, such as activating mutations [150, 152]. PROTACs offer great potential to overcome the RT resistance of tumor cells by targeting, for example, receptors like EGFR, androgen (AR) and estrogen receptor (ER), or kinases, including CDKs and mitogen-activated protein kinase 1/2 (MEK ½; reviewed in [153]). Promising data for the development of PROTACs to sensitize tumors in vivo have further been acquired in a xenograft model using the FDA-approved AR degradation enhancer ASC-J9, showing significant effects in prostate cancer [154]. The development of orally active PROTACs ARV-110 and ARV-471 targeting the AR and the ER receptor, respectively, has further facilitated their implementation in clinical trials [155, 156]. For instance, ARV-110 is currently being tested as a treatment for metastatic castration-resistant prostate cancer in phase I and phase II studies and ARV-471 is being similarly explored in breast cancer. The first preliminary efficacy findings for both compounds look promising, underlining the great potential of PROTACs [155].

## Nanoparticles to improve on delivery of molecular targeted therapies

A valuable approach to reduce unwanted effects and increase the efficacy of molecular targeted strategies is the development of nanotechnology-based platforms tailored to the patients’ profiles, with superior drug-carrier capabilities and improved accumulation at the tumor site. Nanoparticles (NP) encompass a huge range of solid colloidal particles with a size between 1 and 1000 nm. Nanotechnology-based platforms offer great potential to increase the efficacy of delivery, solubility, stability, and cellular uptake of molecular targeted substances, chemotherapeutics, small molecules, or nucleic acids [157, 158]. This is facilitated by enhancing their accumulation in the tumor, protection from degradation, and their selective response to the environment for controlled release [159]. Furthermore, their small size enables an enhanced permeability and retention (EPR) effect in tumors, thus enhancing NP accumulation [160]. This accumulation is based on the unique pathophysiological characteristics of tumors, such as extensive angiogenesis and subsequent chaotic vascular architecture [160]. Another critical function of NPs is that their surface can be functionalized with a large variety of different molecules, such as proteins, peptides, affinity agents, e.g., antibodies or aptamers, lipids, carbohydrates, and folic acid, as well as different fluorescent dyes [159, 161–163]. Such functionalization improves the tumor- or tumor microenvironment-specific delivery as well as finding the specific target of interest.

Furthermore, protection of pharmaceutical ingredients loaded on or into the NPs such as nucleic acids, proteins, or peptides has a major impact on therapeutic efficacy, as many of these agents would otherwise undergo rapid enzymatic degradation in the human circulation [161]. Based on their formulations, recent nanotechnologies can be grouped into different classes: inorganic, liposomal or polymeric NPs, carbon nanotubes, extracellular vesicles (EVs), DNA/RNA nanotechnology, and nanoplatforms for photodynamic/photothermal therapy [7, 158, 164, 165]. Inorganic nanocarriers, mainly based on metals, have been evaluated as therapeutic and diagnostic agents [158, 166, 167]. By this, inorganic NPs such as superparamagnetic iron oxide nanoparticles (SPIONs), which are composed of high atomic number (Z) elements, have been intensively investigated as radiosensitizers and are now clinically available. These NPs promote their RT-sensitizing effect through physical interaction with IR, amplifying the effects of RT by reactive oxygen species (ROS) generation, scattering, and enhanced generation of secondary electrons. So far, gold and other metallic NPs have been extensively analyzed in preclinical studies, but with only limited success in terms of clinical translation. By contrast, hafnium oxide NPs (NBTXR3, Nanobiotix) have been explored in patients with soft tissue sarcoma of a locally advanced stage, showing in both a phase I and II/III setting improved rates of pathological complete responses combined with RT as compared to RT alone (16% vs. 8%) [168, 169] and are now being tested in several clinical settings [165].

Liposomal nanocarriers are the most-studied NPs for molecular targeted therapy. They cover colloidal vesicles with a double lipid membrane composed of amphiphilic phospholipids, allowing the encapsulation of drugs, nucleic acids, or small molecules in their hydrophobic membrane or core. Advantages cover their biocompatibility, low toxicity and immunogenicity, and small size [157], while disadvantages include limited drug-loading capacity, rapid release of loading, and rapid degradation. Polymeric carrier systems are based on different structures including dendrimers (hyperbranched macromolecules), or polymeric micelles (amphiphilic core/shell). Polymeric nanocarriers display superior stability in vivo, enhanced blood circulation time as well as loading efficacy, and facilitate a drug release which can be more controlled as compared to liposomes [157, 158]. Extracellular vesicles (EVs) are endogenous particles surrounded by a bilayer of phospholipids secreted by eukaryotic and prokaryotic cells and loaded with proteins, nucleic acids, lipids, and metabolites for intercellular communication. EVs have different sizes and have been classified into small EVs (sEVs; < 100 to ~ 200 nm) and medium/large EVs (> 200 nm up to 2000 nm), with the latter known also as microvesicles and, in case of cell death, apoptotic bodies. Modifications enable the incorporation of therapeutic agents, and EVs are considered as excellent nanocarriers due to their stability and biocompatibility [164]. RNA molecules also possess great potential as nanocarriers for targeted delivery of siRNAs due to their structural and functional versatility [121]. RNA NPs can further carry RNA aptamers specific for receptors on the surface of tumor cells, acting as ligands to promote receptor-mediated endocytosis [122]. Finally, photodynamic therapy takes advantage of combining a photosensitizer drug and visible or laser light to produce ROS for enhanced killing of tumor cells. Similarly, photothermal therapy involves near-infrared (NIR) laser/light to induce hyperthermia in the tumor site for thermal ablation of malignant cells. These effects can be enhanced by NP-based delivery of photosensitizers or photothermal agents using metallic, metal oxide, or organic NPs. Both therapeutic options can be combined with nanocarriers loaded with anti-cancer drugs or siRNAs [170, 171].

In addition to a plethora of preclinical studies describing advanced NPs loaded with drugs, proteins, RNA, or small molecules, and carrying different surface modifications for targeted delivery and chemosensitization, a number of reports also describe NP-mediated molecular targeting for RT sensitization (reviewed in [158, 165, 172]). For instance, one study describes sensitization of NSCLC by cetuximab-conjugated mesoporous silica NPs targeting EGFR and loaded with polo-like kinase 1 (Plk1) siRNA [173]. Apart from these mainly preclinical reports, implementing NP-mediated delivery of drugs and molecular targeting agents in clinical trials remains challenging, as the pharmacokinetics in animal studies often do not reflect the efficacy and safety in humans. Nevertheless, NPs hold great potential for the transport of new classes of therapeutics, such as nucleic acids, but more detailed investigations are required [158].

## Conclusion

Although several approaches to enhancing RT or CRT in tumors using molecular targeting tools have been studied for a long period of time, their application and translation in the clinical setting is still challenging. These shortcomings can likely be attributed to the lack of suitable biomarkers to select patients for specific treatment approaches based on their oncogenic drivers. Another explanation might be the multifaceted response to RT/CRT, where back-up pathways working alongside the targeted one hamper efficient sensitization of the tumor. In this context, increased efficacy can be obtained by using multiple molecular targeting agents concomitantly [70].

While many strategies have not yet found their way into clinical evaluation, with further progress, RNA- or protein-degrading PROTAC-based targeting, for example of DDR and signal transduction, may in the future allow for development of innovative therapeutic strategies with improved RT/CRT sensitization [174]. Finally, the rapid and promising developments in the nanotechnology field create another way to improve on molecular targeted therapy delivery of regular CT, small molecules, RNA-based therapy, and photodynamic therapy, thus reducing side effects and increasing tumor targeting.
